# Spa therapy interventions for post respiratory rehabilitation in COVID-19 subjects: does the review of recent evidence suggest a role?

**DOI:** 10.1007/s11356-021-15443-8

**Published:** 2021-07-17

**Authors:** Maria Chiara Maccarone, Stefano Masiero

**Affiliations:** 1 Physical Medicine and Rehabilitation School, University of Padova, Via Giustiniani 3, 35128 Padua, Italy; 2 Rehabilitation Unit, Department of Neuroscience, University of Padova, Padua, Italy

**Keywords:** SARS-CoV-2, Health resort medicine, Pulmonary recovery, Balneotherapy

## Abstract

Pulmonary rehabilitation is essential in post-COVID subjects, reporting respiratory impairment after the discharge from the hospital. Because the number of patients with respiratory outcomes is high and there are few facilities available, we wonder if a spa setting could represent a valid out-of-hospital alternative. We aim to explore recent evidence related to respiratory rehabilitation in the spa environment to understand if it can represent an appropriate setting for respiratory rehabilitation interventions in post-COVID subjects. Studies were found by screening PubMed, MEDLINE, and Google Scholar databases from 2011 up to February 2021. Studies were eligible if they were reviews, randomized controlled trials (RCTs), or clinical trials, investigating respiratory interventions in the spa environment. Recent evidence has shown that inhalations and mineral-rich water immersions are effective in fighting and preventing multiple chronic respiratory tract diseases. Therefore, these treatments could also be applied to post-COVID patients with medium long-term respiratory outcomes.

## Introduction

In late December 2019, the novel coronavirus-2019 (COVID-19) pandemic emerged in Wuhan, China, and soon spread exponentially to more than 200 countries around the globe. At present, 169,597,415 confirmed cases have been reported (World Health Organisation 2021).

Although the infection can manifest with symptoms affecting different systems, the major concern is related to the involvement of the respiratory system. Indeed, although in most cases (80%) the infection proceeds asymptomatically or with mild symptoms, 13.8% of cases have severe disease and 6.1% are critical and require hospitalization in the Intensive Care Unit for significant respiratory involvement (Vermund and Pitzer 2020). Respiratory symptoms include dyspnea, reduced blood oxygen saturation, and respiratory failure, requiring mechanical ventilation primarily in patients with comorbidities such as obesity, diabetes mellitus, ischaemic heart disease, cancer, and chronic obstructive pulmonary disease (COPD) (Siddiq et al. 2020). Patients with mechanical ventilation may also develop acute respiratory distress syndrome (ARDS), pulmonary oedema, atelectasis, and pulmonary embolism with right-sided heart failure (Boyer et al. 2015).

In these patients, early pulmonary rehabilitation treatments can enhance physical outcomes and Quality of Life (QoL) (Brown et al. 2019). The 2013 American Thoracic Society (ATS)/European Respiratory Society (ERS) Statement defines pulmonary rehabilitation as “a comprehensive intervention based on a thorough patient assessment followed by patient-tailored therapies, which include, but are not limited to, exercise training, education, and behaviour change, designed to improve the physical and psychological condition of people with chronic respiratory disease and to promote the long- term adherence of health-enhancing behaviors” (Spruit et al. 2013).

Given the possibility of long-term disabling outcomes involving the cardiorespiratory domain, post-hospitalization pulmonary rehabilitation may be considered in all patients hospitalized with COVID-19 (Wang et al. 2020), also after the resolution of the acute infection. In post-discharge survivors of COVID-19 infection, restrictive ventilation disorders, compromised diffusion capability, and residual impaired lung function have been reported, particularly in elderly patients (You et al. 2020).

Spas can offer rehabilitation interventions for patients with musculoskeletal and neurological disabilities alone or in association with other traditional thermal therapies (Masiero et al. 2020a, b, Bernetti et al. 2020). Since it is necessary to start respiratory treatments as soon as possible, continuous reorganization hospitals and rehabilitation activities are needed (Bai et al. 2021), and there are limited facilities available to treat patients in the post-acute period (Maccarone and Masiero 2021), we aim to explore recent evidence related to pulmonary rehabilitation in the spa environment in order to understand if it can represent an appropriate setting for respiratory rehabilitation interventions in post-COVID subjects.

## Material and methods

A literature update was conducted with the aim of searching for recent evidence of spa therapy interventions’ effectiveness in respiratory disorders.

Screening of PubMed, MEDLINE, and Google Scholar databases from 2011 up to February 2021 was performed by MCM in order to find studies. The keywords chosen were spa therapy, balneotherapy, Health resort medicine, inhalations, respiratory rehabilitation, and pulmonary rehabilitation. The Boolean Logic has been used to produce various configurations of search strings.

Studies were eligible if they were reviews, randomized controlled trials (RCTs), or clinical trials. Only studies on human subjects and written in English were considered.

## Results

### Inhalations

Spa treatments specifically targeting the respiratory tract, which include inhalations, aerosol, and nasal showers, are recommended in the treatment of respiratory disorders. They have a well-known therapeutic and preventive effect, stimulating the immune responses and the resistance of the respiratory tract to pathogenic microorganisms. This positive effect is generally based on the patients’ subjective sense of wellbeing, whereas is more difficult to quantify clinical improvements (Corradi et al. 2012).

Inhalations involve inhaling water particles with a diameter of more than 20 μm that reach the mucosa of the nasopharynx and larynx. Therefore, they are indicated in diseases of the upper respiratory tract, such as rhinitis, sinusitis, chronic pharyngitis, and laryngitis.

Sulphur-rich water inhalation has been demonstrated in COPD patients to increase muco-ciliary clearance, decrease the synthesis of pro-inflammatory cytokines and the inflammatory mucosal infiltration, and reduce the levels of elastase produced by the neutrophils. Also, treatment with inhaled salt-bromide-iodine thermal water has been shown to have a mild anti-inflammatory effect on the airways in COPD patients (Khaltaev et al. 2020). Inhalation therapy with salsojodic mineral waters has vasodilating activity on the bronchial mucosa and increases the production of secretory IgA and muco-ciliary clearance (Khaltaev et al. 2020).

In chronic inflammatory diseases of the upper airways nonresponsive to pharmacological therapy, a 14-day course of radioactive water warm vapour inhalations followed by nasal aerosol has been demonstrated to be effective in improving the muco-ciliary clearance (Passali et al. 2013).

In elderly subjects affected by chronic rhinosinusitis treated with crenotherapy with sodium chloride sulphate hyperthermal water, 1 month after the treatment the nasal cytological assessment showed statistically significant improvements in the ciliary motility and in the count of neutrophils (Cantone et al. 2014).

Radon-enriched inhalation therapy has been demonstrated to improve objective indicators of nasal functionality in allergic rhinitis and chronic rhinosinusitis and to cause relief of pulmonary obstruction in asthma (Kesiktas et al. 2011). In patients with airflow obstruction (FEV1/FVC < 0.7), the index FEV1/FVC (forced vital capacity) significantly increased after 12 days of inhalation treatments (Corradi et al. 2012).

### Mineral-rich water immersions

The utilization of thermal mineral-rich waters for water immersions is often difficult to study, since it is usually part of the global spa therapy. Physical modalities combined with mineral-rich water immersions have been demonstrated to be associated with significant improvements in dyspnea scale and spirometric measurements in patients affected by fibromyalgia at the end of the treatment and also after 6 months (Passali et al. 2017).

In COPD patients, exercise in thermal water has been demonstrated to be more suitable when compared to gym-based exercises and they could encourage socialization (Khaltaev et al. 2020).

Finally, thermal baths have been shown to improve biochemical parameters in exhaled breath condensate of heavy smokers (Carubbi et al. 2019).

## Discussion

For post-COVID-19 subjects, a comprehensive rehabilitative approach comprising a multidisciplinary and professional team, offering cardiorespiratory, neuromuscular, and psychological interventions, is recommended (Agostini et al. 2021). The main goals of respiratory rehabilitation in post-COVID-19 subjects are to improve symptoms of dyspnea, to reduce chronic inflammation in the airways, to ameliorate chest wall kinematics, to reduce complications and long-term outcomes, to minimize disability, and to improve QoL (Wang et al. 2020, Liu et al. 2020, Antonelli and Donelli 2020).

Since recent evidence has shown that in chronic pathologies of the lower and upper respiratory tract, thermal water treatments are effective in improving respiratory function, the use of spa therapy can be hypothesized also in post-COVID patients with persistent respiratory outcomes.

The spa setting, benefiting also from a multidisciplinary staff, can offer specific breathing physio-kinesiotherapy associated with mineral waters inhalation treatments, focused on the enhancement of respiratory outcomes that could affect COVID-19 survivor (Antonelli and Donelli 2020; Masiero et al. 2020a).

Mineral-rich water inhalations have been demonstrated to be effective in ameliorating the elastic properties of pulmonary interstitium, reducing inflammation, and stimulating the muco-ciliary function. One of the current hypotheses explaining the mechanism of action of inhalation treatments is that inhalations can replace or increase glutathione (GSH), reducing oxidative stress associated with inflammation in lung disorders and production of reactive oxygen species (ROS) (Corradi et al. 2012). The heat applied to the whole body during the water immersions could have ulteriorly positive effects on the respiratory system modulating innate and acquired immune defences (Cohen 2020; Masiero et al. 2020b; Maccarone et al. 2021a; Maccarone et al. 2020).

Spa treatments can also offer psychological benefits and enhance mental wellness, improving relaxation and increasing stress resilience and QoL (Antonelli and Donelli 2020; Masiero et al. 2020a; Cohen 2020).

Moreover, spa setting can represent an appropriate location to take care of comorbidities such as obesity, advanced age, COPD, fatigue, neurological, and musculoskeletal conditions (Masiero et al. 2020a; Masiero 2008; Masiero et al. 2018; Masiero et al. 2019; Maccarone et al. 2021b).

Combining all these beneficial effects, in the spa setting, customized post-COVID programmes could be drawn, referring, as suggested by Antonelli et al. [20], to already existing rehabilitative plans, such as those ones prescribed for work-related respiratory diseases.

Finally, we would like to remark that post-COVID-19 subjects accessing spa treatments must not be infectious anymore, which means that they must have no residual symptoms and two consecutive negative RT-PCR results (Antonelli and Donelli 2020; Masiero et al. 2020a).

## Conclusion

Recent evidence has shown that different treatments using thermal water are effective in several respiratory tract diseases; therefore, spa environment could represent an appropriate out-of-hospital setting for respiratory rehabilitation in post-COVID subject. Further studies are needed to test the effectiveness of respiratory rehabilitation protocols in the spa setting for these patients.
